# LAPTM4B Allele *2 Is a Marker of Poor Prognosis Following Hepatic Tumor Resection for Hepatocellular Carcinoma

**DOI:** 10.1371/journal.pone.0034984

**Published:** 2012-04-03

**Authors:** Hua Yang, Guojun Zhai, Xiaoxu Ji, Fuxia Xiong, Jing Su, Michael A. McNutt

**Affiliations:** 1 Department of Cell Biology, School of Basic Medical Sciences, Peking University, Beijing, China; 2 Department of Interventional Radiology and Vascular Surgery, Peking University Third Hospital, Beijing, China; 3 Department of General Surgery, Peking University Third Hospital, Beijing, China; 4 Department of Pharmacology, Loma Linda University, California, United States of America; 5 Department of Pathology, School of Basic Medical Sciences, Peking University, Beijing, China; National Institute of Environmental Health Sciences, United States of America

## Abstract

**Background:**

Lysosomal protein transmembrane 4 beta (LAPTM4B) is a gene related to hepatocellular carcinoma that has two alleles designated LAPTM4B*1 and LAPTM4B*2. This study aimed to investigate the correlation of LAPTM4B genotype with prognosis and clinicopathologic features in patients who have undergone resection for hepatocellular carcinoma (HCC).

**Methodology/Principal Findings:**

The LAPTM4B genotype was analyzed by PCR in 68 patients who had undergone curative hepatic resection for hepatocellular carcinoma. The correlation of LAPTM4B genotype with clinicopathologic parameters was assessed with the Chi-squared test. Differences in patient survival were determined by the Kaplan–Meier method. Multivariate analysis of prognostic factors was carried out with Cox regression analysis. Patients with LAPTM4B *2 had both significantly shorter overall survival (OS) and shorter disease-free survival (DFS) (both P<0.001). Multivariate analysis showed that LAPTM4B genotype is an independent prognostic factor for OS and DFS (both P<0.001).

**Conclusions/Significance:**

Allele *2 of LAPTM4B is a risk factor associated with poor prognosis in patients with resected HCC. LAPTM4B status may be useful preoperatively as an adjunct in evaluation of the operability of HCC.

## Introduction

Hepatocellular carcinoma (HCC) is the third most common cause of cancer death in the world and is the most common cause of cancer death in China [1]–[4]. There are many risk factors which are associated with HCC and contribute to transformation of liver cells. These factors include alcohol, hepatitis B and C virus and conditions such as cirrhosis [5], [6]. However these risk factors for the most part have not been of clinical value in predicting patient survival or in evaluating operability of HCC. It would therefore be beneficial for selection of therapy in these patients to find markers which predict prognosis in HCC.

Lysosomal protein transmembrane-4 beta was originally cloned from HCC in our laboratory, and this gene resides at 8q22.1 and contains seven exons [7]. Previous studies have demonstrated that LAPTM4B mRNA and protein are significantly up-regulated in a wide variety of cancers such as lung cancer, gallbladder carcinoma, extra-hepatic cholangiocarcinoma, cervical carcinoma, colon cancer and ovarian cancer [8]–[13]. The LAPTM4B gene is amplified in many breast cancers and its amplification is related to breast carcinoma recurrence [14]. Moreover, transfection of LAPTM4B cDNA results in overexpression of LAPTM4B which is associated with promotion of HCC cell invasion in nude mice xenografts, as well as with other malignant phenotypic features such as cell proliferation and migration, and multidrug resistance [15]–[18]. Conversely knockdown of LAPTM4B by RNA interference reverses multiple malignant phenotypes [16]. These observations and data argue that this gene plays a fundamental role in many types of neoplasia, and previous studies have shown that LAPTM4B protein is overexpressed in HCC, and this overexpression is significantly correlated with tumor histopathological grade and prognosis. This has led us to consider whether it could serve as a prognostic marker in patients under evaluation for resection of HCC.

The LAPTM4B gene has two alleles designated LAPTM4B*1 and LAPTM4B*2 (GenBank No. AY219176 and AY219177). Allele *1 contains only one copy of a 19-bp sequence, whereas this sequence is duplicated and tandemly arranged in allele *2 in the first exon of the LAPTM4B gene [19]–[24]. As reported in our previous study, we found that the allelic frequencies of the LAPTM4B*2 allele were 38.24% in the HCC group and 24.07% in the control group, representing a significant different between these two groups (P<0.001), and suggesting that the LAPTM4B*2 allele is associated with significantly increased risk of hepatocellular carcinoma [23]. The existence of two LAPTM4B gene variant alleles raises the possibility that prognosis in HCC may be related to gene polymorphism. The present work therefore aimed to investigate whether there is a correlation of LAPTM4B gene polymorphism with prognosis in HCC patients who have undergone surgical resection. In order to investigate this relationship, we evaluated the genotype of patients who have had HCC surgery for attempted curative resection, and our results show that LAPTM4B allelic variation is significantly associated with prognosis in these patients. LAPTM4B genotype may therefore be a marker for HCC prognosis and might serve as an adjunct marker for preoperative evaluation.

## Materials and Methods

### Patients

Blood samples were obtained from 68 HCC patients who were hospitalized and underwent surgical resection in Third Hospital Affiliated to Peking University from January 2002 to December 2009. All patients who had attempted curative resection for HCC in Third Hospital during this period were included in this study and there were no other selection criteria for inclusion other than availability of follow up information.

There are 57 male patients and 11 female patients in this study, which is not unexpected as M∶F ratios in areas of high HCC incidence may be as high as 8∶1 (Robbins Basic Pathology, 7th edition, 2003), and the mean patient age was 51.7 (range 29–80). TNM staging was carried out for each patient according to AJCC-UICC guidelines (AJCC_UICC, 5th edition, 1997), and clinical hepatic status was determined according to standard criteria (Liver Pathology, Churchill Livingstone Publishing House, New York, 1986).

Patient clinicopathologic features, which include history of hepatic viral infection, size of tumor, presence or absence of portal vein invasion, histopathologic tumor differentiation, and serum AFP level, together with TNM stage and presence or absence of tumor recurrence are summarized (Table 1). The Institutional Ethics Committee of Peking University approved this study before investigation was begun, and every patient gave informed consent for participation.

**Table 1:** Relationship between LAPTM4B genotypes and clinicopathological features of HCC.

Variables	Patients	LAPTM4B genetype			*P^a^*
		^*^1/1	^*^1/2	^*^2/2	
Gender					0.172
Male	57	16	32	9	
Female	11	6	3	2	
Age (years)					0.415
<65	44	14	21	9	
≥65	24	8	14	2	
Viral status					0.171
Hepatitis virus B	49	16	22	11	
Hepatitis virus C	12	3	9	0	
Both hepatitis virus B and C	2	0	2	0	
Non-B, Non-C	5	3	2	0	
Tumor size					0.852
<5 cm	37	13	18	6	
≥5 cm	31	9	17	5	
portal vein invasion					0.001
No	49	22	22	5	
Yes	19	0	13	6	
Histopathological differentiation					0.009
WD	20	12	6	2	
MD	25	8	14	3	
PD	23	2	15	6	
Serum AFP level					0.144
<25 ng/ml	35	14	18	3	
≥25 ng/ml	33	8	17	8	
TNM stage					<0.001
I–II	23	18	4	1	
III–IV	45	4	31	10	
Recurrence					<0.001
No	17	12	5	0	
Yes	51	10	30	11	

LAPTM4B, Lysosomal protein transmembrane 4 beta; HCC, hepatocellular carcinoma; *a*, Chi-square test.

### DNA extraction

Human peripheral blood samples were collected in EDTA tubes and kept at −20°C. Blood was thawed in a water bath at room temperature and genomic DNA was isolated from 200 ul of each blood sample using the NucleoSpin Blood genomic DNA extraction kit (Macherey-Nagel, Duren, Germany) for PCR assay.

### PCR analysis

LAPTM4B genotype was determined by PCR analysis using the specific primers F (forward): 5′ GCCGACTAGGGGACTGGCGGA 3′ (nt 72–92) and R (reverse): 5′ CGAGAGCTCCGAGCTTCTGCC 3′ (nt 255–275). PCR assay was carried out in a 20 ul reaction mixture containing 0.5 U Taq DNA polymerase (NEB, Beijing, China) using 1 ul of template DNA of 100 ng/ul. The PCR conditions were 95°C denaturation for 5 min, 35 cycles of 30 s at 94°C, 30 s at 60°C and 30 s at 72°C, followed by extension at 72°C for 10 min. PCR products were analyzed by electrophoresis in a 2% agarose gel and visualized with ethidium bromide.

### Statistical analysis

The Chi-square test was used to demonstrate differences in the genotypic distribution of LAPTM4B and categorical variables. The Kaplan–Meier method and log-rank test were used to evaluate differences in patient overall survival and disease-free survival. Disease-free survival was defined as the interval from the time of surgery until any sign of tumor recurrence or metastasis was found during patient follow up clinical examination. Potential prognostic factors were subjected to multivariate analysis using Cox regression analysis (Proportional hazard model). SPSS10.0 software (SPSS Inc., Chicago, IL) was used for all statistical analysis. P values<0.05 were defined as statistically significant.

## Results

### Genotypes of the LAPTM4B gene

**Figure 1:**
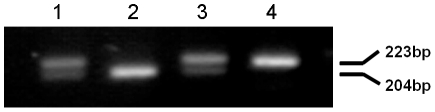
LAPTM4B genotyping. Analysis by separation with 2% agarose gel electrophoresis. Lanes 1, 3: genotype*1/2; lane 2: genotype*1/1; lane 4: genotype *2/2.

Three different genotypic LAPTM4B polymorphisms designated LAPTM4B*1, LAPTM4B*2 and LAPTM4B*1/*2 were identified by PCR assay. As shown in Figure 1, genotypes *1/1 is represented by a 204-bp band, *2/*2 is represented by a 223-bp band, and genotype *1/*2 shows both of these bands. All bands were identified with PCR using specific primers and separated by electrophoresis on a 2% agarose gel.

### Genotypes of LAPTM4B and clinical parameters

These 68 patients who underwent hepatic resection at Third hospital affiliated with Peking University from 2002 to 2009 were followed clinically for periods ranging from 2 to 92 months (average 33 months). At the end date for follow up (December 31, 2010), 16 patients (23.5%) were alive, and 52 (76.5%) had died of disease. We found that genotype *2 of the LAPTM4B gene was significantly associated with recurrence, poor histopathologic differentiation, higher TNM stage and portal vein invasion (Table 1; P<0.05), but not with viral infection status, tumor size or serum AFP level, age or gender (Table 1; P>0.05).

### LAPTM4B genotype and HCC prognosis

**Figure 2:**
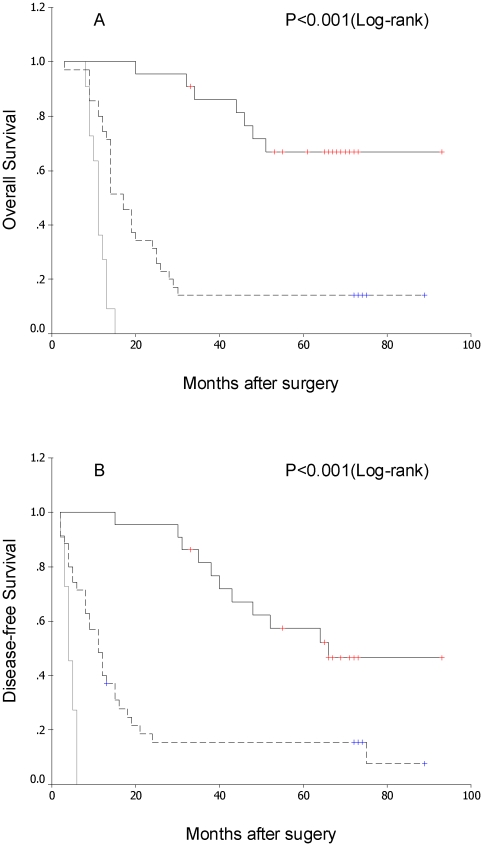
Comparison of survival of patients with HCC after surgery resection based on evaluation of LAPTM4B genotypes. LAPTM4B genotype *1/1, black solid line; LAPTM4B genotype *1/2, black dashed line; LAPTM4B genotype *2/2, grey solid line. (A) Overall survival after surgery. (B) Disease-free survival after surgery.

Using the Kaplan–Meier method and the log-rank test, genotype *2 of the LAPTM4B gene showed correlation both with shorter disease-free survival and shorter overall survival in the 68 patients who underwent curative resection for HCC (Fig. 2A and B, and Table 2; both P<0.001). In addition, as expected survival benefit was also found in patients with lower TNM stage, lower grade histopathologic differentiation and absence of portal vein invasion both for overall and disease-free survival (Table 2; P<0.05). No other clinicopathologic features showed predictive value in this analysis (Table 2; P>0.05).

**Table 2:** Univariate Survival Analysis of OS and DFS in 68 patients with HCC.

Variables	No. of cases	OS			DFS		
		Mean±SE (month)	95% CI	*P ^a^*	Mean±SE (month)	95% CI	*P ^a^*
Gender				0.476			0.403
Male	57	39±5	(30–48)		31±5	(22–40)	
Female	11	38±7	(24–51)		35±7	(21–49)	
Age(years)				0.456			0.769
<65	44	38±5	(28–48)		33±5	(22–43)	
≥65	24	38±6	(27–50)		32±6	(20–44)	
Viral status				0.182			0.359
Hepatitis virus B	49	37±5	(28–47)		30±5	(21–39)	
Hepatitis virus C	12	40±10	(20–60)		35±11	(14–57)	
Both hepatitis virus B and C	2	19±10	(0–37)		9±4	(2–15)	
Non-B, Non-C	5	62±11	(40–83)		54±11	(33–76)	
Tumor size				0.665			0.749
<5 cm	37	41±6	(30–52)		34±6	(23–45)	
≥5 cm	31	38±6	(26–50)		32±6	(19–44)	
portal vein invasion				<0.001			<0.001
No	49	51±5	(41–61)		43±5	(33–53)	
Yes	19	12±1	(10–15)		7±1	(5–10)	
Histopathological differentiation				0.003			0.005
WD	20	57±8	(41–73)		47±8	(32–62)	
MD	25	37±5	(26–47)		32±6	(21–43)	
PD	23	23±5	(13–32)		16±5	(6–26)	
Serum AFP level				0.233			0.208
<25 ng/ml	35	45±6	(33–57)		38±6	(26–49)	
≥25 ng/ml	33	31±4	(22–39)		25±5	(15–34)	
TNM stage				<0.001			<0.001
I–II	23	73±7	(60–86)		64±7	(51–77)	
III–IV	45	23±3	(17–30)		17±4	(11–24)	
LAPTM4B genotypes				<0.001			<0.001
^*^1/1	22	75±6	(64–86)		66±6	(55–78)	
^*^1/2	35	27±4	(18–35)		21±5	(12–30)	
^*^2/2	11	11±1	(10–12)		4±0	(4–5)	

LAPTM4B, Lysosomal protein transmembrane 4 beta; HCC, hepatocellular carcinoma; OS, overall survival; DFS, disease-free survival; *a*, Log-rank test.

### LAPTM4B genotype is an independent prognostic maker in patients who have undergone curative resection for HCC

Multivariate Cox regression analysis revealed that LAPTM4B genotype (RR, 3.147, 95%CI, 1.619–6.115, P = 0.001) and portal vein invasion (RR, 3.542, 95%CI, 1.752–7.158, P<0.001) are independent prognostic markers for overall survival of patients with HCC (Table 3; P<0.05). LAPTM4B genotype (RR, 2.729, 95%CI, 1.427–5.220, P = 0.002) and portal vein invasion (RR, 2.953, 95%CI, 1.437–6.068, P = 0.003) are also independent prognostic markers for disease-free survival of patients with HCC (Table 3; P<0.05).

**Table 3:** Multivariate Survival Analysis of OS and DFS in 68 patients with HCC.

Variables	OS			DFS		
	RR	95%CI	*P^a^*	RR	95%CI	*P^a^*
TNM stage	2.482	(1.003–6.143)	0.049	1.905	(0.868–4.181)	0.108
Portal vein invasion	3.542	(1.752–7.158)	<0.001	2.953	(1.437–6.068)	0.003
Histopathological differentiation	1.325	(0.861–2.037)	0.200	1.336	(0.888–2.010)	0.165
LAPTM4B genotype	3.147	(1.619–6.115)	0.001	2.729	(1.427–5.220)	0.002

LAPTM4B, Lysosomal protein transmembrane 4 beta; HCC, hepatocellular carcinoma; OS, overall survival; DFS, disease-free survival; RR, relative risk; CI, confidence interval; *a*,Cox regression test.

## Discussion

In this study, we tested LAPTM4B genotype by PCR assay in 68 patients who had surgical resection for hepatocellular carcinoma. We found that LAPTM4B *2 is significantly correlated with recurrence, higher TNM stage and invasion of the portal vein, but this allele showed no relationship to patient liver status, histopathologic tumor grade, viral status, size of tumor, serum AFP level gender or patient age. Moreover, patients with LAPTM4B *2 (alleles *2/2 or *1/2) had significantly poorer overall survival (OS) and disease- free survival (DFS) as compared to patients with LAPTM4B *1/1.

This is the first time correlation of LAPTM4B genotype both with prognosis and clinicopathologic features has been demonstrated in hepatocellar carcinoma. This association of genotype with clinicopathologic findings and prognosis is consistent with the putative role LAPTM4B plays in carcinogenesis and tumor progression. Previous studies which have shown upregulation and/or amplification of the LAPTM4B gene in a variety of human cancers, and this together with experimental evidence showing malignant phenotypic features can be induced or reversed by transfection or knockdown respectively of LAPTM4B strongly suggests that this gene plays a fundamental role in the neoplastic mechanism(s) of these tumors [17], [25], [26]. There are some recent findings which may in part serve to illustrate how LAPTM4B functions in tumors which harbor this gene. For example, overexpression of LAPTM4B may activate the AKT signaling pathway and proto-oncogenes including c-fos, c-myc, and c-jun and thus promote malignant transformation. In addition, knock-down of LAPTM4B or mutation of the PPRP motif in the N-terminal region of LAPTM4B attenuates its roles in tumorigenesis and metastasis [26].

The allele LAPTM4B*1 (GenBank No. AY219176) differs from allele LAPTM4B*2 (GenBank No. AY219177) in that it contains a particular single 19-bp sequence, whereas LAPTM4B *2 contains two of these sequences in a tight tandem array in the 5′ UTR of exon 1. The allelic frequencies of the *2 allele were 33.9%, 33.2%, and 40.1% in individuals with gastric, colon, and lung cancer, respectively, which were significantly higher than those of corresponding healthy controls [21], [22]. LAPTM4B*1 produces a 35 kD protein while LAPTM4B*2 produces a 40 kD protein with an extra 53 amino acids at the N terminus which are not present in the protein produced by LAPTM4B*1. This difference in proteins is due to the 19-bp difference in the first exon of the gene which alters the ORF. Previous studies have shown that LAPTM4B polymorphisms are associated with increased risk for gastric cancer and cervical cancer in Chinese patients. In view of the latter and given the extensive evidence of LAPTM4B's involvement in neoplasia, as well as its demonstrated relation to histologic grade and tumor recurrence in some cancers, it is likely that this 19-bp sequence plays an important role in genetic transcriptional regulation or that the extra 53 amino acids at the N terminus produced by LAPTM4B* 2 may influence physiological activity and function in tumor cells.

Hepatocellular carcinoma is a major health problem, and is the leading cause of death from cancer worldwide. Although the exact molecular mechanisms which underlie the function of LAPTM4B in hepatocarcinogenesis have not as yet been worked out, LAPTM4B*2 may be an oncogene or play an important role in cell cycle control. However, LAPTM4B *2 is significantly associated with tumor recurrence, poor histopathologic differentiation, higher TNM stage and presence of portal vein invasion which argues that it is a driver of hepatocellular carcinoma and therefore warrants further investigation. In addition, it is an independent prognostic factor for postoperative survival in patients who have undergone curative surgical resection for HCC. It is likely that the relatively high overall failure rate in surgical intervention for HCC is at least in part due to imprecision in preoperative staging. Therefore, it is possible that LAPTM4B *2 will be useful as an adjunct for evaluation of HCC prior to embarking upon curative surgical resection.
